# Perioperative management of emergency cesarean delivery in a pregnant woman with uncorrected diabetic ketoacidosis: a case report

**DOI:** 10.1186/s40981-025-00832-6

**Published:** 2025-12-29

**Authors:** Satoshi Naruse, Takaki Kasai, Momoka Kojima, Hiroshi Ueda, Chieko Akinaga, Yoshiki Nakajima

**Affiliations:** 1 Perinatal Center, Hamamatsu University School of Medicine, 1-20-1 Handayama, Chuo-Ku, Hamamatsu, Shizuoka 431-3192 Japan; 2 Department of Anesthesiology and Intensive Care, Hamamatsu University School of Medicine, 1-20-1 Handayama, Chuo-Ku, Hamamatsu, Shizuoka 431-3192 Japan; 3 Department of Medical Education, Hamamatsu University School of Medicine, 1-20-1 Handayama, Chuo-Ku, Hamamatsu, Shizuoka 431-3192 Japan

**Keywords:** Obstetrical anesthesia, Cesarean delivery, Diabetes mellitus, Diabetic ketoacidosis, Insulin infusions, Perioperative care, Pregnancy

## Abstract

**Background:**

Diabetic ketoacidosis (DKA) during pregnancy is a rare but life-threatening complication associated with high fetal mortality. Prompt metabolic stabilization is essential, although emergency cesarean delivery may be required. Reports on anesthetic management are limited.

**Case presentation:**

A 24-year-old primigravida with poorly controlled type 2 diabetes presented at 35 weeks of gestation with nausea and non-reassuring fetal status (NRFS). DKA was confirmed using laboratory tests. Emergency cesarean delivery was performed for NRFS before complete stabilization. Metabolic management was conducted by endocrinologists and anesthesiologists. Combined spinal-epidural anesthesia (CSEA) was intraoperatively administered by anesthesiologists with real-time hemodynamic and metabolic management guided by arterial blood gas analysis using an arterial line while continuing insulin and glucose therapy. Postoperatively, metabolic derangements resolved, and the mother and neonate were discharged without complications.

**Conclusions:**

This case demonstrates that emergency cesarean delivery for uncorrected DKA can be achieved using CSEA, timely hemodynamic support, and coordinated metabolic management.

## Background

Diabetic ketoacidosis (DKA), a serious complication of diabetes mellitus characterized by hyperglycemia, ketonemia, and metabolic acidosis, causes significant morbidity/mortality [1]. Pregnancy increases susceptibility to hormonal insulin resistance, enhances lipolysis, and accelerates ketogenesis, particularly during late gestation [2]. A Chinese case–control study reported DKA incidence rates of 8.9%/3.1% in pregnant/non-pregnant hospitalized women with diabetes [3]. A UK population-based study estimated incidences of 16.6/1,000 and 1.1/1000 births with type 1 and 2 diabetes [4]. Common contributing factors for DKA in pregnancy include protracted vomiting/starvation, infections, poor glycemic control or insulin omission, insulin pump malfunction, β-sympathomimetic agents, and corticosteroid therapy [4–9]. Maternal outcomes are favorable, with mortality rate of < 1%, possibly due to improved access to medical care and longer hospital stay [10, 11]. However, fetal mortality remains high (16–28%) [4, 7, 12]. When a non-reassuring fetal status develops, cesarean delivery may be required before correcting maternal metabolic derangements [13]. Therefore, anesthesiologists must manage severe acidosis, unstable hemodynamics, and rapidly fluctuating insulin requirements intraoperatively. Detailed reports on the anesthetic and metabolic management of cesarean deliveries in uncorrected DKA are rare. Herein, we present a case successfully managed using combined spinal-epidural anesthesia (CSEA), real-time arterial blood gas guidance, and interdisciplinary collaboration.

## Case presentation

A 24-year-old nulliparous woman (height 153 cm, weight 84.2 kg, pre-pregnancy weight 65 kg, body mass index [BMI] 36.0 kg/m^2^) with pregestational type 2 diabetes mellitus. One year pre-pregnancy, she was hospitalized for severe hyperglycemia (glucose, 300 mg/dL, hemoglobin A1c [HbA1c], 13%) with ketonuria, after improvement on insulin, she was switched to oral antihyperglycemic therapy, and self-discontinued follow-up. At 9 weeks’ gestation, she was referred to our hospital (HbA1c, 6.3%). Basal–bolus insulin was initiated (detemir four units at bedtime and aspart six units before meals). Home logs showed pre-meal 170–190 mg/dL and 2-h postprandial 200–300 mg/dL. Insulin requirements increased progressively, reaching detemir 54 units at bedtime and aspart 38 units before each meal by late pregnancy.

**Fig. 1:**
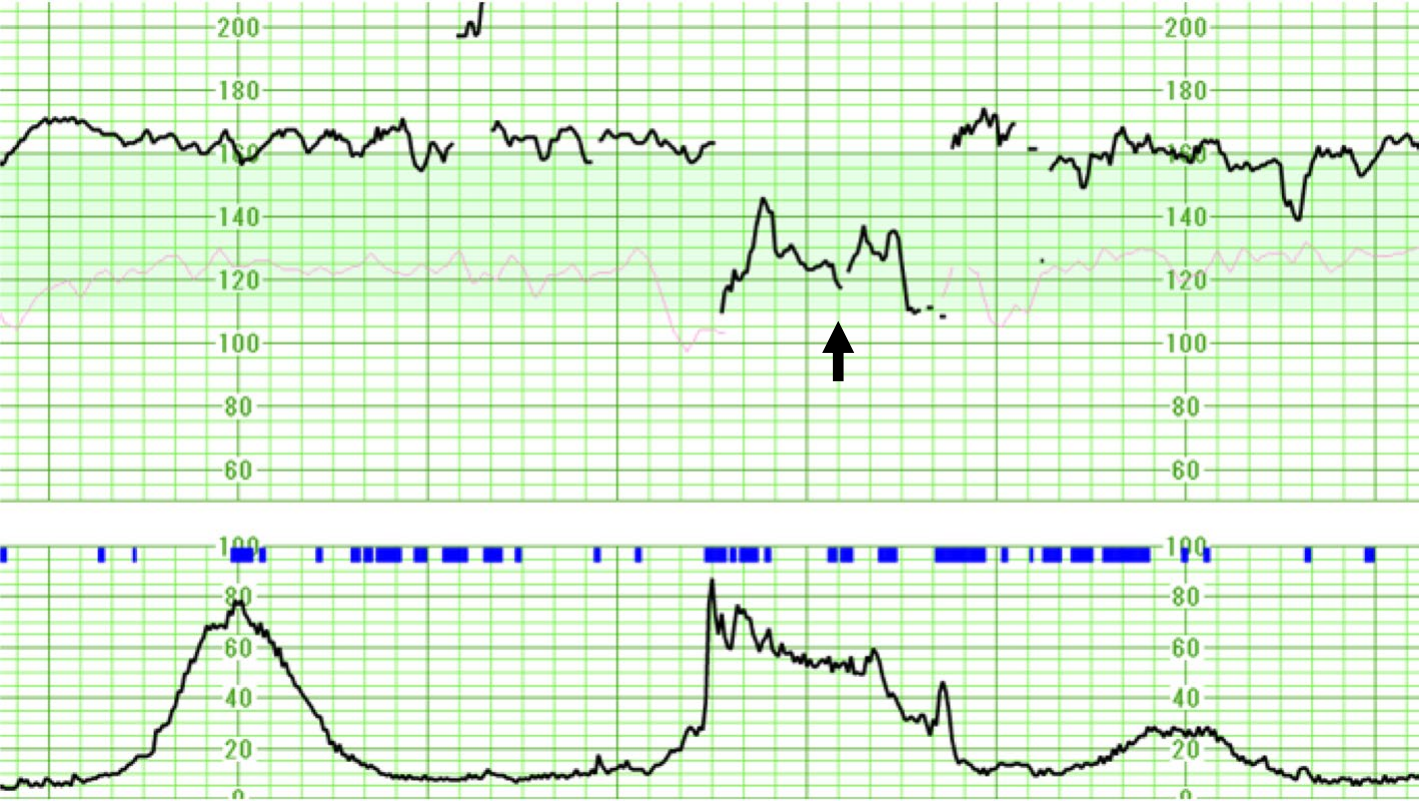
Cardiotocography on hospital admission showing fetal tachycardia with reduced variability. Cardiotocography upon hospital admission showing fetal tachycardia (approximately 160 beats/min), moderately reduced variability (6–25 beats/min), and recurrent variable decelerations (arrows). Acceleration was not observed. These findings led to a diagnosis of a non-reassuring fetal status

At 35 weeks’ gestation, she presented to an obstetric outpatient clinic with worsening nausea. Fetal monitoring showed tachycardia (160 beats/min), moderately reduced variability, and recurrent variable decelerations, consistent with non-reassuring fetal status (NRFS) (Fig. 1). Emergency admission was conducted due to the risk of cesarean delivery.

Vitals on admission were: temperature, 36.0 °C; blood pressure, 112/84 mmHg; heart rate 92/min, respiratory rate 20/min with Kussmaul respiration; and percutaneous oxygen saturation (SpO₂), 98% on room air. DKA was confirmed by laboratory tests (glucose, 455 mg/dL; pH, 7.24, bicarbonate level, 10.9 mmol/L, and 2 + ketonuria). Emergency cesarean delivery was planned before full metabolic stabilization.

Endocrinologists initiated a continuous human insulin infusion (2U/h), accompanied by a 10-unit bolus of insulin aspart/glucose infusion at 4 g/h. Fluid resuscitation with 0.9% saline was initiated at 1,000 mL/h for one hour, followed by 100 mL/h. The endocrinology team remained involved in intraoperative metabolic management and collaborated with anesthesiologists throughout the intraoperative/postoperative periods.

**Fig. 2:**
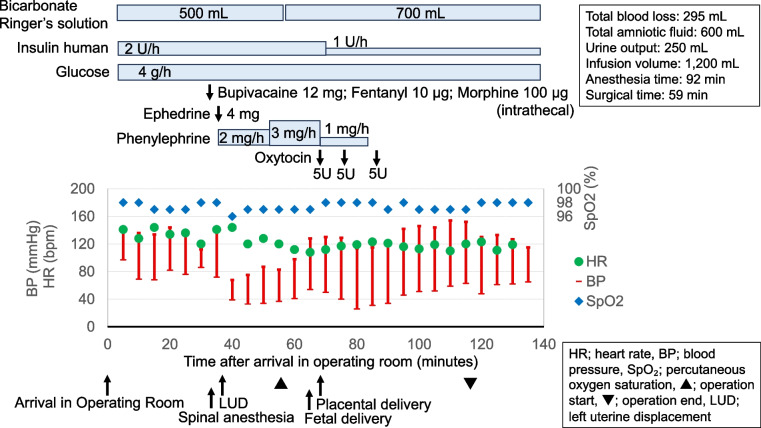
Perioperative hemodynamics and pharmacological interventions during cesarean section under uncorrected diabetic ketoacidosis. The upper panel shows the treatment time course. The lower panel shows the trends in the vital signs. Key clinical events were annotated along the x-axis timeline as follows: arrival in the operating room, spinal anesthesia, onset of left uterine displacement, delivery, and placental delivery

At arrival in the operating room, approximately 2 h after the decision, her heart rate was 130/min, blood pressure 144/98 mmHg, and SpO₂ 98%. A radial arterial line was used for continuous monitoring and blood sampling. CSEA was performed with an epidural catheter placed at L2–L3 to facilitate supplemental dosing and spinal anesthesia at L4–L5 (12 mg hyperbaric bupivacaine, 10 µg fentanyl, and 100 µg morphine). Prophylaxis against post-spinal hypotension included continuous phenylephrine infusion (2–3 mg/h), a rapid 500 mL bicarbonate-based crystalloid bolus administered preoperatively, and left uterine displacement. However, systolic blood pressure decreased to 68 mmHg, requiring prompt correction with an ephedrine bolus. Surgical incision was initiated after confirming bilateral sensory block up to T4. Insulin infusion was continued at 2U/h and crystalloids were titrated according to urine output, blood pressure, heart rate, and arterial waveform. The neonate, a 2,456 g female, had Apgar scores of 9/10 and an umbilical artery pH of 7.17. Insulin resistance was expected to improve after placental delivery, and the infusion rate was reduced to 1 U/h. At skin closure, arterial blood gas (ABG) analysis revealed a pH, 7.29; hemoglobin, 12.3 g/dL; and hematocrit, 37.6%. with only mild improvement in metabolic acidosis (Table 1). The total intraoperative infusion volume was 1,200 mL. Intraoperative anesthesia was adequate, without requiring epidural boluses, and the catheter was removed immediately postoperatively (Fig. 2).

**Table 1:** Perioperative arterial blood gas and laboratory values

	Hospital admission	OR admission	At skin closure	2 h postoperative	6 h postoperative	12 h postoperative
pH	7.24	7.27	7.29	7.26	7.35	7.36
PaCO₂ (mmHg)	10.6	11.8	14.8	22.8	30.1	30.5
PaO₂ (mmHg)	–	125	133	98.6	81.5	81.8
Bicarbonate (mmol/L)	10.9	5.4	7	10.1	16.4	17.1
Base excess (mmol/L)	–	−21.4	−19.6	−14.7	−8.2	−7.1
Sodium (mmol/L)	128	136	136	137	138	138
Potassium (mmol/L)	5.1	4.1	4.1	3.9	3.3	3.3
Lactate (mmol/L)	–	1.9	1.6	1.1	0.9	0.7
Glucose (mg/dL)	455	340	331	311	261	243

*OR* Operating room; “–,” not available in the medical records

The patient was admitted to the intensive care unit postoperatively. Intravenous insulin (1U/h) and glucose (4 g/h) were continued with supplemental potassium chloride (1.5 mEq/h). To address osmotic diuresis, 2,300 mL of lactated Ringer’s solution was administered within 12 h. ABG analysis was performed every 2–4 h to guide therapy. Analgesia included intrathecal morphine and intravenous acetaminophen (1 g every 6 h for 24 h). Thromboprophylaxis with subcutaneous enoxaparin (2,000 IU every 12 h) was continued for 6 days. By postoperative day (POD) 1, the pH rose to 7.34 and the glucose level decreased to 261 mg/dL (Table 1). On POD 2, insulin was transitioned to a subcutaneous regimen and oral intake resumed. The patient was discharged on POD 7 without complications.

The neonate was admitted to the neonatal intensive care unit (NICU). Initial venous blood gas showed pH 7.16, bicarbonate 18.2 mmol/L, lactate 5.6 mmol/L, and glucose 131 mg/dL, consistent with metabolic acidosis. Follow-up measurements 2 h later showed spontaneous resolution (pH 7.23, bicarbonate 25.4 mmol/L, lactate 2.2 mmol/L, and glucose 114 mg/dL). The neonate remained stable and was discharged on day 18 without complications.

## Discussion

This case highlights the challenges in managing emergency cesarean delivery in a pregnant patient with uncorrected DKA. Despite severe acidosis, successful neuraxial anesthesia and metabolic management were achieved through close interdisciplinary collaboration. The favorable maternal and neonatal outcomes emphasize the importance of timely obstetric decision-making and individualized anesthetic planning in high-risk settings.

Consistent with the established risk factors for DKA during pregnancy [4–9], this case is explainable by specific precipitants, with DKA likely triggered by late-gestation insulin resistance with high insulin requirements, persistent hyperglycemia, reduced intake due to acute nausea, and possibly suboptimal adherence.

In maternal DKA with fetal compromise, emergency cesarean delivery may be necessary before full correction, as fetal hypoxia and acidemia can worsen rapidly [12]. Therefore, anesthesiologists must prepare to manage both hemodynamic instability and severe acid–base disturbances with fluctuating insulin requirements. Herein, fetal tachycardia and reduced variability prompted the decision to proceed with cesarean delivery before metabolic stabilization, consistent with prior reports supporting immediate delivery in cases of deteriorating fetal status due to uncorrected DKA [14]. This situation reflects the clinical dilemma that, while maternal outcomes benefit from metabolic stabilization preoperatively, fetal survival and intact neurological prognosis may depend on expeditious delivery unless the gestational age precludes viability.

There are no definitive guidelines on the choice between neuraxial and general anesthesia for cesarean deliveries in DKA [15]. Neuraxial anesthesia is generally preferred because it avoids airway manipulation [16], reduces fetal exposure to anesthesia [17, 18], and allows patient consciousness during delivery. In parturients with diabetes, the risk of aspiration is increased by gastroparesis [19], supporting the use of neuraxial techniques; however, metabolic acidosis, autonomic dysfunction, and dehydration may exacerbate hypotension induced by sympathetic blockade [20, 21]. General anesthesia is indicated when altered consciousness, such as in hyperglycemic coma, requires airway protection. Therefore, the choice of modality should be individualized.

In this case, obesity and a short fasting interval increased the risk of difficult airway management; however, the urgency permitted safe neuraxial anesthesia, and CSEA was selected. Although standard prophylaxis for postspinal hypotension was administered, the patient experienced transient hypotension, suggesting that reduced intrathecal bupivacaine with epidural supplementation, allowing additional local anesthetic administration through the epidural catheter, may improve hemodynamic stability in similar cases [22]. A relatively small crystalloid preload of 500 mL may be insufficient, especially with preexisting dehydration [23].

Current guidelines, consensus recommendations, and a contemporary review for DKA management emphasize early and comprehensive correction of metabolic derangements [24–27]. Therapeutic goals include plasma ketone < 0.6 mmol/L, pH ≥ 7.30, bicarbonate ≥ 18 mmol/L [24], and plasma glucose 150–200 mg/dL to limit osmotic diuresis [25]. The recommended treatment is intravenous insulin infusion; once glucose approaches the target, dextrose-containing fluids should be added to allow insulin therapy continuation to suppress ketogenesis [24–27]. Fluid resuscitation began with isotonic saline at 1–2 L/h for 1–2 h [24–27]. Electrolytes, particularly potassium levels, must be closely monitored [24–27]. After placental delivery, insulin resistance decreases rapidly, requiring a half-to-one-third reduction in infusion rate [27]. Additionally, pharmacological thromboprophylaxis is recommended [24, 25]; this case was managed accordingly [24–27].

The neonate exhibited transient metabolic acidosis and lactate elevation that resolved within 2 h, suggesting that intrauterine compromise due to maternal acidosis and dehydration improved promptly post-delivery. Previous studies have reported increased risks of NICU admission, neonatal hypoglycemia, and metabolic acidosis in infants born to mothers with DKA [7, 28]. The absence of complications herein may be attributable to timely obstetric intervention and effective perioperative management.

Emergency cesarean delivery in patients with uncorrected DKA can be performed safely when supported by guideline-based metabolic management, neuraxial anesthesia, and interdisciplinary collaboration. Real-time adjustment of insulin/fluid therapy guided by ABG analysis facilitated favorable maternal/neonatal outcomes, highlighting the importance of coordinated and proactive management in high-risk obstetrics.

## Data Availability

All the relevant data have been included in this article. Further details are available from the corresponding author on request.

## Abbreviations

DKA: Diabetic ketoacidosis
NRFS: Non-reassuring fetal status
CSEA: Combined spinal-epidural anesthesia
BMI: Body mass index
HbA1c: Hemoglobin A1c
SpO₂: Percutaneous oxygen saturation
ABG: Arterial blood gas
POD: Postoperative day
NICU: Neonatal intensive care unit
